# Characterization of *p53* From the Marine Crab *Portunus trituberculatus* and Its Functions Under Low Salinity Conditions

**DOI:** 10.3389/fphys.2021.724693

**Published:** 2021-10-21

**Authors:** Xianyun Ren, Lei Wang, Yao Xu, Qiong Wang, Jianjian Lv, Ping Liu, Jian Li

**Affiliations:** ^1^Key Laboratory for Sustainable Utilization of Marine Fisheries Resources, Ministry of Agriculture, Yellow Sea Fisheries Research Institute, Chinese Academy of Fishery Sciences, Qingdao, China; ^2^Function Laboratory for Marine Fisheries Science and Food Production Processes, Qingdao National Laboratory for Marine Science and Technology, Qingdao, China; ^3^Jiangsu Key Laboratory of Marine Bioresources and Environment/Jiangsu Key Laboratory of Marine Biotechnology, Jiangsu Ocean University, Lianyungang, China

**Keywords:** *Portunus trituberculatus*, apoptosis, P53, low salinity stress, RNA interference

## Abstract

*Portunus trituberculatus*, or the swimming crab, is tolerant of reduced salinity; however, the molecular mechanism of this tolerance is not clear. Cells can be damaged by hyperosmotic salinity. The protein p53, sometimes referred to as “the guardian of the genome,” displays versatile and important functions under changing environmental conditions. Herein, the *P. trituberculatus p53* gene (designated as *Ptp53*) was cloned and studied. The full-length *Ptp53* cDNA comprised 1,544bp, with a 1,314bp open reading frame, which encodes a putative polypeptide of 437 amino acids. Quantitative real-time reverse transcription PCR assays revealed ubiquitous expression of *Ptp53* in all tissues examined, with the gills showing the highest expression level. Extensive apoptosis was detected under low salinity conditions using terminal deoxynucleotidyl transferase nick-end-labeling staining. Oxidative stress was induced under low salinity conditions, consequently leading to apoptosis. Low salinity stress caused significant upregulation of Ptp53 mRNA and protein levels in the gills. Moreover, compared with that in the control group, the mortality of *Ptp53*-silenced crabs under low salinity stress was enhanced significantly. Taken together, our findings suggest that Ptp53, *via* regulation of apoptosis and antioxidant defense, played important functions in the low salinity stress response of the swimming crab.

## Introduction

Salinity is one of the most important environmental factors affecting the distribution and physiological activities of aquatic organisms (Huang et al., 2019). Organisms often experience stress resulting from changes in environmental salinity, although there are differences among species. The ion regulation mechanisms of freshwater and marine species have been studied extensively, for example, low salinity induced mRNA expression levels of *Na^+^-K^+^-ATPase*, *V-type H^+^-ATPase*, and *Diuretic Hormone* in the orange mud crab *Scylla olivacea* (Rahi et al., 2020); the ion transport-related genes *chloride channel protein 2* and *ABC* were significantly downregulated in mud crab *Scylla paramamosain* under high salinity (Zhang et al., 2020); and however, only a few studies have focused on the role of apoptosis. Changes in cell contact with the outside environment and ion transport depend on the external osmotic pressure, which is affected to the greatest extent by changes in salinity. Adaptation to salinity is a well-known trigger of apoptotic mechanisms, especially in the chloride (or mitochondrial) cells of earthworms and the epidermal components of the skin and gastrointestinal tract of fish (Ching et al., 2013). In crustaceans, the gills experience direct exposure to the external aqueous environment and thus comprise the main site at which ion movement is balanced between gain and loss; therefore, the gills are the main site of apoptosis during salinity adaptation in aquatic animals (McNamara and Faria, 2012).

Apoptosis has important functions in tissue and organ differentiation, as well as the removal of terminally damaged cells (Jones, 2001). Various signaling pathways regulate biochemical events and apoptosis in cells (Edinger and Thompson, 2004), causing certain cellular changes, such as chromosomal DNA fragmentation, chromatin condensation, nuclear fragmentation, shrinkage, and blebbing, ultimately causing the death of cells (Norbury and Zhivotovsky, 2004; Elmore, 2007). P53, a tumor suppressor, is a vital regulator that mediates cells’ response to many stress signals. Acting as a transcription factor, p53 functions in DNA damage repair, energy metabolism, apoptosis, and cell cycle regulation. Therefore, during cell stress, p53 is vital for the functions of associated signaling networks (Cheng et al., 2021). The alterations of salinity induce physiological stress, which is closely related to the generation of reactive oxygen species (ROS) can cause oxidative stress (Kim et al., 2017). In response to ROS generation, superoxide dismutase (SOD) decomposes superoxide anion to hydrogen peroxide, and catalase (CAT) decomposes hydrogen peroxide to oxygen and water (Truong et al., 2018). Under oxidative stress, p53 has particularly important functions (Sablina et al., 2005). Oxidative stress can be reduced *via* p53-mediated increases in the expression of antioxidant enzyme genes, including *glutathione peroxidase* (*GPX*) and *MnSOD* (Bakthavatchalu et al., 2009). Other genes, such as *BAX* (encoding BCL2-associated X and apoptosis regulator) and *BCL2* (encoding BCL2 apoptosis regulator), are trans-activated by p53 (Hussain et al., 2004).

There have been several studies related to p53 in aquatic animals. For example, host antiviral defense in *Siniperca chuatsi* critically involves activated p53 (Guo et al., 2017). In addition, the *p53* transcriptions in hepatopancreas of *Takifugu obscurus* were significantly upregulated after *Vibrio parahaemolyticus* infection (Cheng et al., 2016). Studies also demonstrated that ambient stress responses are greatly affected by p53 (Qi et al., 2013; Sun et al., 2016). However, our knowledge regarding p53’s function in crustaceans remains limited.

The commercially important aquaculture species *Portunus trituberculatus* (the swimming crab) is distributed widely in the coastal waters of China, Taiwan, Japan, and Korea (Dai et al., 1977). During its cultivation, *P. trituberculatus* is frequently subject to substantial salinity fluctuations, with potentially significant consequences for its yield and productivity (Lv et al., 2013). Salinity, an environmental factor closely related to osmotic pressure, has a significant effect on the respiratory metabolism, growth, survival, and immune defense of *P. trituberculatus* (Sun et al., 2019). During their breeding season, wild swimming crabs must swim from the estuarine region back into the sea (Chen et al., 2019). Commonly, low salinity conditions are used to cultured swimming crabs in artificial ponds; however, drought or heavy rain can alter the salinity, with consequent detrimental effects on mortality and productivity. Compared with wild females, pond-reared female swimming crabs’ ovaries mature poorly, which inhibits the sustainable development of crab farming (Wu et al., 2010).

The aim of the present study was to investigate the effects of low salinity stress on apoptosis and oxidative stress in *P. trituberculatus*. Thus, we cloned and characterized the full-length p53 cDNA sequence from *P. trituberculatus* (named *Ptp53*). In addition, we examined the Ptp53 mRNA and protein expression under conditions of low salinity. Finally, RNA interference (RNAi) was used to analyze Ptp53’s role in the response to low salinity. The findings of this study will increase our understanding of the functions of *P. trituberculatus* p53 in the response to low salinity conditions.

## Materials and Methods

### Ethical Statement

All animal experiments were conducted in accordance with relevant national and international guidelines and were approved by the Yellow Sea Fisheries Research Institute. In China, catching wild shrimp from seawater does not require specific permits. Our study did not involve endangered or protected species.

### Specimens

The swimming crabs, *P. trituberculatus* at 80days age (32±8g in body weight), were obtained from a local farm in Qingdao, China. All the crabs were acclimated in the laboratory (33ppt, 18°C) for 1week before the experiment. The water quality was maintained at salinity of 33ppt with ammonia-N<0.5mgL^−1^, nitrite <0.10mgL^−1^ and dissolved oxygen (DO)>5mgL^−1^ at pH 7.0–9.0.

### Low Salinity Stress and Sampling

According to our previous method, we conducted a pre-experiment of salinity stress and calculated the low salinity level as 11ppt; a design experiment was conducted, which allowed us to calculate that the salinity causing 72h half-fatality in the 80day old crabs was 11ppt (Gao et al., 2019; Sun et al., 2019). The salinity experiments were set up using two different levels of salinity: The 11ppt group and the 33ppt group (control) with three replicates (*n*=42 crabs). Six crabs from each replicate were sampled randomly at 0, 3, 6, 12, 24, and 48h after low salinity stress.

### Cloning of p53

Rapid amplification of cDNA ends (RACE) was used to clone the full-length p53 cDNA from *P. trituberculatus* using a SMARTer^®^ RACE cDNA Amplification Kit (Takara, Shiga, Japan), as described previously (Li et al., 2019). The specific primers designed using conserved expressed sequence tag sequences for p53 are listed in Table 1.

**Table 1:** Nucleotide sequences of the PCR primers used in this study.

Primers	Sequences (5' - 3')	Purpose
*p53*-F1	TCAGTTCCCCTTCACCATCCTCC	3’-RACE
*p53*-F2	GATGGAGCCTGGAACAGAAAACC	3’-RACE
*p53*-R1	AGGATGGTGAAGGGGAACTGACA	5’-RACE
*p53*-R2	GCTGAGGATGAAACTGCGGCTGA	5’-RACE
*UPM-long*	CTAATACGACTCACTATAGGGCAAGCA	RACE-universal primers
	GTGGTATCAACGCAGAGT	
*UPM-short*	CTAATACGACTCACTATAGGGC	RACE-universal primers
*NUP*	AAGCAGTGGTATCAACGCAGAGT	RACE-universal primers
*Ptp53-RNAi*-F	GGUACCACACGAUAGAGUUTT	RNAi
*Ptp53-RNAi*-R	AACUCUAUCGUGUGGUACCTT	RNAi
*GFP-RNAi*-F	TAATACGACTCACTATAGGGTGGAGTGGTCCCAGTTCTTGTTGA	RNAi
*GFP-RNAi*-R	TAATACGACTCACTATAGGGGCCATTCTTTGGTTTGTCTCCCAT	RNAi
*p53*-F	GAGGATGAAACTGCGGCTGA	qRT-PCR
*p53*-R	AACTCTGTCCCTCCCACTAC	qRT-PCR
*CuZnSOD*-F	GCGGTAGTGAACTTTGTGCC	qRT-PCR
*CuZnSOD*-R	GAATGTTGCCAAGGTCTCCA	qRT-PCR
*CAT*-F	ATGAGCAGGCAGAGAAGTGG	qRT-PCR
*CAT*-R	TCAAGTGTGATGCGACCAAC	qRT-PCR
*GPX*-F	GTCCTGGTAACAACTTTGAGCC	qRT-PCR
*GPX*-R	ATGATACACTTGGGGTCTGCC	qRT-PCR
*Bcl-2*-F	TCCTCCATAGCGTCCCTTACCT	qRT-PCR
*Bcl-2*-R	CCAGCAGGGATTTCTAAGGAC	qRT-PCR
*Bax*-F	GGTTAGGATAAAGGGAGAGGA	qRT-PCR
*Bax*-R	CAGCACATCGGTAAAGGAAGT	qRT-PCR
*caspase-3*-F	TTCCCAGTATCTCTGTCGTG	qRT-PCR
*caspase-3*-R	TTCCAGTAAATCATAGCGG	qRT-PCR
*β-actin*-F	CGAAACCTTCAACACTCCCG	qRT-PCR
*β-actin*-R	GGGACAGTGTGTGAAACGCC	qRT-PCR

### Bioinformatic Analysis

The sequences were identified using BLAST searching at the National Center for Biotechnology Information.^1^ The protein functional domains were analyzed using SMART.^2^ Open reading frame (ORF) finder^3^ was used to deduce the ORF and the putative encoded protein sequence. ExPASy^4^ was used to predict the theoretical isoelectric point (pI) and molecular mass. DNAman software was used for multiple sequence alignment, and the Gene Tool software was used to analyze the nucleotide and deduced protein sequences. The AMCA web server^5^ was used to identify antibacterial peptide sequences. The MEGA6.0 software was used to construct a phylogenetic tree *via* the neighbor-joining method. The SignalP 4.1 Server^6^ was used to detect signal peptide sequences.

### TUNEL Assay

Terminal deoxynucleotidyl transferase nick-end-labeling (TUNEL) staining in gill tissue was performed using a *In Situ* Cell Death Detection Kit (Roche, Basel, Switzerland) according to the manufacturer’s protocol. Briefly, tissue sections were deparaffinized and rehydrated before being digested using proteinase K for 30min. The TUNEL reaction mixture was added to the sections and incubated at 37°C in a humidified chamber for 1h. Sections then were washed in phosphate-buffered saline (PBS), stained with 3,3'-diaminobenzidine (DAB), counterstained with Mayer-hematoxylin, observed under a microscope, and photographed. Staining was quantified using Image-Pro Plus 6.0 (Media Cybernetics, Rockville, MD, United States).

### Enzyme Assays

#### Supernatant Preparations

One hundred milligrams of gill sample (0.1g) were subjected to homogenization in ice-cold buffer comprising 20mm Tris-HCl (pH 7.6), 10% (v:v) glycerol, 1.0mm dithiothreitol, and 1.5mm EDTA at 0°C. After removing the debris by centrifugation at 12,000×*g* (4°C, 5min), the supernatants were harvested by centrifugation at 3000×*g* (4°C, 25min) to analyze the activities of SOD, CAT, and caspase-3, and the protein contents were determined.

#### Assaying GPX, CAT, and SOD Activities

Organs were excised from each crab, weighed, placed in phosphate buffer solution (pH 7.2) at a ratio of 1:9 (w/v), and then homogenized on ice. The SOD, CAT, and GPX activities in the supernatant were detected as described previously (Rotruck et al., 1973; Nishikimi, 1975; Bradford, 1976; Sun et al., 1988; Góth, 1991; Wang et al., 2011).

#### Caspase-3 Activity

A Caspase-3 Assay Kit (Shanghai Enzyme-linked Biotechnology Co., Ltd., Shanghai, China) was used to measure caspase 3 activity following the manufacturer’s protocol. Briefly, the reaction system containing 60μl of 2mm substrate Ac-DEVD-pNA, 100μl of supernatant (0.1mgml^−1^), and 140μl of reaction buffer was maintained at 37°C for 4h. Free pNA produces a yellow color that was quantified using a microtiter plate reader (SpectraMax 190, Molecular Devices, San Jose, CA, United States) at 405nm.

### Quantitative Real-Time Reverse Transcription PCR

For qRT-PCR, RNAs were extracted from samples using Trizol according to the manufacturer (Roche, San Francisco, CA, United States). Single-stranded cDNAs were generated using HiScript II Q RT SuperMix for Quantitative real-time PCR (qPCR; +gDNA wiper) kit (Vazyme, Jiangsu, China). qPCR was then performed on a Applied Biosystems^™^ 7,500 Real-Time PCR instrument (ABI, Foster City, CA, United States; Livak and Schmittgen, 2001) using the ChamQ SYBR qPCR Master Mix (High ROX Premixed) kit (Vazyme). Primers for *Ptp53* and *β-actin* (internal control) are shown in Table 1. The thermal cycling conditions were as follows: 10min at 95°C; followed by 40cycles of 95°C for 30s and 60°C 34s; 95°C for 5s; 60°C for 1min; and 95°C for 15s. The expression of the gene relative to the control was assessed using the standard 2^−ΔΔCt^ method (Livak and Schmittgen, 2001).

### Western Blotting

To explore its function, levels of Ptp53 protein were measured using Western blotting according to our previously published method (Ren et al., 2021). Thirty micrograms of protein from each sample was separated using 15% SDS-polyacrylamide gel electrophoresis, followed by electrotransfer to polyvinylidene fluoride membranes (Genscript, Nanjing, China). TBST buffer (0.05% Tween 20, 0.15M NaCl, and 10mm Tris-HCl, pH 8.0) with 3% skimmed milk was used to block the membranes, followed by overnight incubation with a polyclonal antibody recognizing p53 (1:1000, Genscript). The membrane was then incubated with horseradish peroxidase-linked anti-rabbit IgG (Beyotime, Jiangsu, China). Development was performed using a DAB Horseradish Peroxidase Color Development Kit (Beyotime). The band intensities from each blot were quantified using an Amersham Imager 600 instrument (GE Healthcare, Chicago, IL, United States). The image gray value was analyzed by ImageJ software, and the expression level of the target protein was reflected by the ratio of the gray value of the target protein band that of the β-actin band.

### RNAi on the Mortality of Crab After Low Salinity Stress

The function of Ptp53 was investigated using small interfering RNA (siRNA). Table 1 shows the primers for p53 and green fluorescent protein (GFP, control). An *in vitro* T7 Transcription kit for siRNA synthesis (Takara) was used to synthesize dsRNA following the manufacturer’s protocol. The 20μl rection system produced 10μg of *Ptp53* dsRNA. In the experimental group, crabs (*n*=20) were injected with various doses of *Ptp53* dsRNA (1μg/g crab weight) into the arthrodial membrane of the fifth swimmeret. In the GFP dsRNA and PBS group, the same concentration of GFP dsRNA or PBS was injected into crabs (*n*=20). At 24h after injection, the crabs were exposed to 11ppt salinity. During the RNAi experiment, dead crabs were collected each hour and not feed during the experiment. In each group, the cumulative crab mortality was determined at 0, 3, 6, 12, 24, and 48h after low salinity stress.

### Statistical Analysis

All values are expressed as the mean±SD. All experimental data were subjected to analysis using SPSS software version 19.0 (IBM Corp., Armonk, NY, United States). Statistical evaluation of the raw data was performed using one-way ANOVA followed by Tukey’s multiple range test. A values of *p*<0.05 was considered statistically significant.

## Results

### Analysis of the Predicted Protein of p53

The *Ptp53* cDNA sequence comprises 1,544bp, containing an ORF of 1,314bp that encodes a putative protein of 437 amino acids (GenBank Accession No. MH155954). The cDNA sequence has a 168bp 5' untranslated region (UTR) and a 230bp 3'-UTR (Supplementary Figure S1). The ExPASy ProtParam analysis showed that the putative Ptp53 protein has a pI of 5.47 and molecular weight of 49.6kDa. Its instability coefficient was 51.61. The p53 amino acid sequence of *P. trituberculatus* showed 56 and 55% similarity with those of *Penaeus vannamei* and *Penaeus monodon*, respectively. The amino acid sequence of *p53* is highly conserved, especially in the amino acid 160–347 region (Supplementary Figure S2). The phylogenetic tree showed that *p53* of *P. trituberculatus* is classified with p53 proteins from vanabin-containing prawns, and the kinship is recent relative to other invertebrates (Supplementary Figure S3).

### p53 Expression in Different Tissues

qRT-PCR was used to analyze the relative mRNA expression of *Ptp53* in various tissues (Supplementary Figure S4). *Ptp53* was expressed constitutively in the heart, hemocytes, cutex, muscle, stomach, hepatopancreas, and gill (order of expression and low to high). The expression of *p53* was significantly higher in the gill than in the other tissues (*p*<0.05).

### TUNEL Assay Results

**Figure 1:**
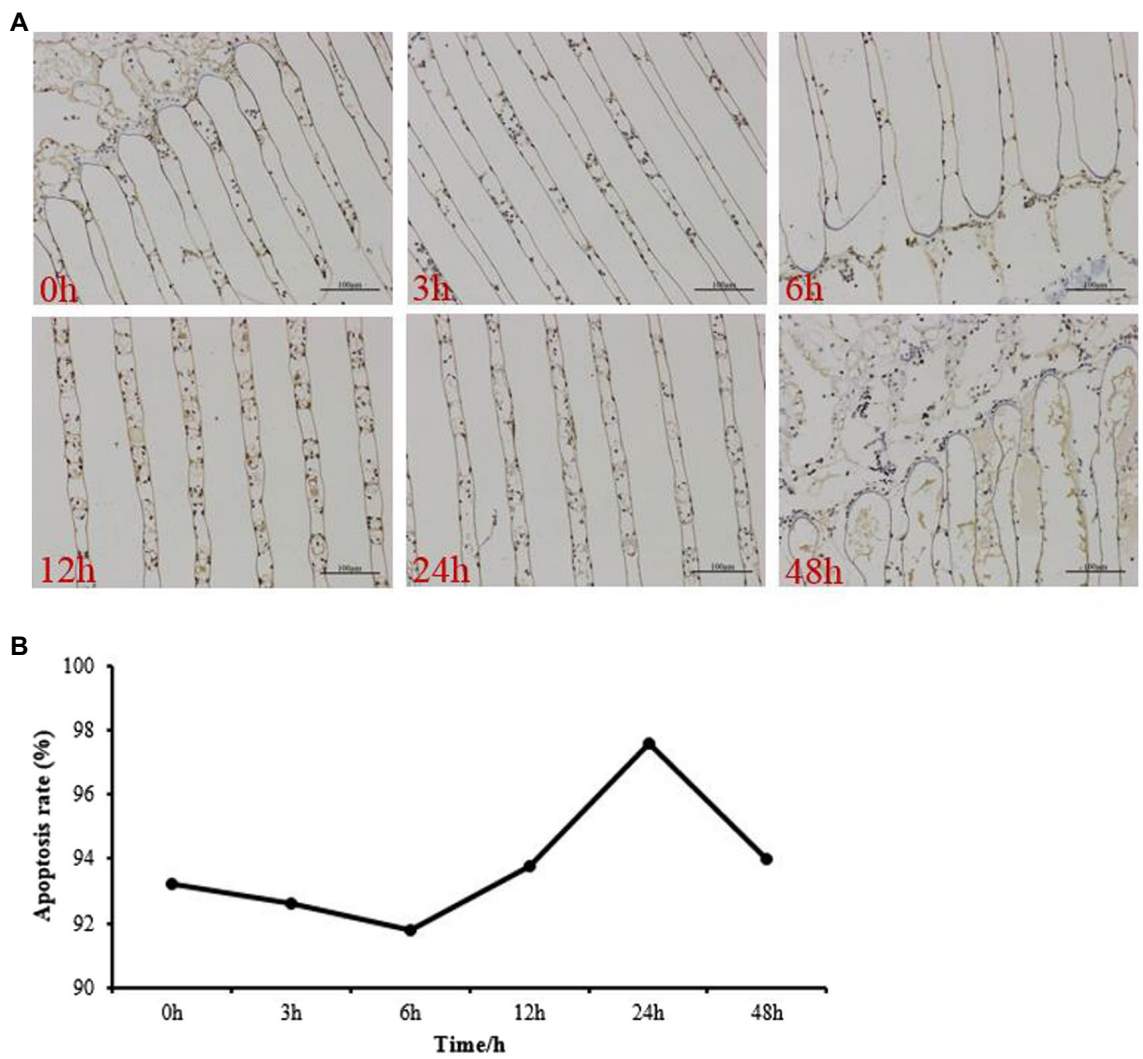
Apoptotic cells in the gills of *Portunus trituberculatus* at 0h, 3h, 6h, 12h, 24h, and 48h by terminal deoxynucleotidyl transferase nick-end-labeling *in situ* immuno-marker assay. Micrograph of a gill tissue sections under 200×magnification **(A)** and the apoptosis rate **(B)**. Bar=100μm.

Figure 1A shows micrographs of gill sections at 200×magnification. The TUNEL assay was used to detect apoptosis, and the nucleus of an apoptotic cell was solidified and brown with a circular, crescent, or irregular shape. The rate of apoptosis was calculated as the number of positive cells/total cells×100. The degree of apoptosis rate in the gill showed a tendency to increase from 0 to 24h and then decrease at 48h, and was significant higher than the control group (Figure 1B).

### Effects of Low Salinity on SOD, CAT, GPX, and Caspase-3 Activities

**Figure 2:**
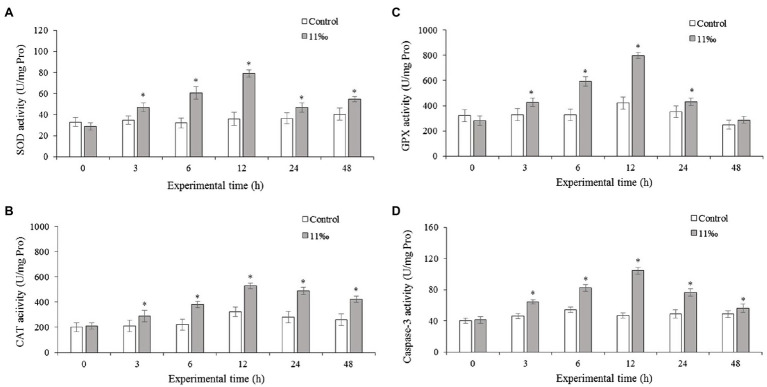
Enzyme activities of SOD **(A)**, CAT **(B)**, GPX **(C)**, and caspase-3 **(D)** in the gills of *P. trituberculatus* under low salinity stress (*n*=6). Data are shown as the mean±SD (*n*=3). Results that are significantly different from the control are indicated using asterisks (*p*<0.05).

To investigate, the effect of low salinity in the activation of the antioxidant and apoptosis pathway was evaluated in the different treatments. The results showed that low salinity had a significant effect on antioxidant and apoptosis enzyme activities (Figure 2). The activities of SOD (Figure 2A) and CAT (Figure 2B) increased significantly after 3h of low salinity stress, and peaked at 12h, then decreased significantly (*p*<0.05) at 24h and 48h in 11ppt group. Similarly, GPX (Figure 2C) and caspase-3 (Figure 2D) activities showed significant increases from 3h to 24h of low salinity stress, and then the GPX activity decreased to control level, while the caspase-3 activity still higher when compared with the control group at 48h.

### Effects of Low Salinity on Antioxidant-Related Genes Expression

**Figure 3:**
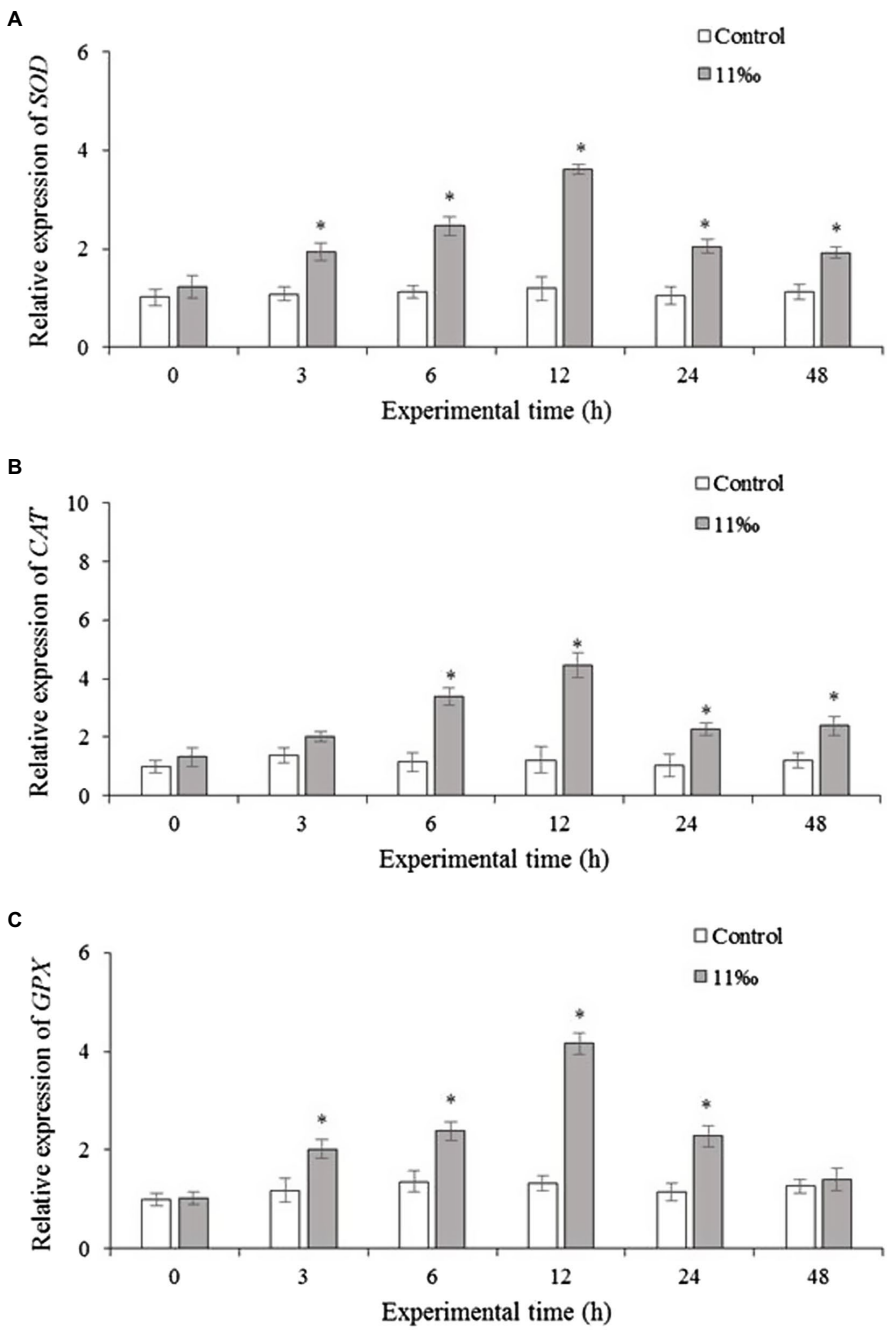
Relative expression levels of *SOD* **(A)**, *CAT* **(B)**, and *GPX* **(C)** in the gills of *P. trituberculatus* after low salinity stress (*n*=6). Results that are significantly different from the control are indicated using asterisks (*p*<0.05). Sod, superoxide dismutase; Cat, catalase; and Gpx, glutathione peroxidase.

The mRNA levels of *Sod*, *Cat*, and *Gpx* were examined in the gills of *P. trituberculatus* under acute salinity stress were shown in Figure 3. Results showed that *Sod* and *Cat* mRNA levels in the gills increased after low salinity treatment (Figures 3A,B). From 6 to 24h of low salinity treatment, *Gpx* mRNA expression increased significantly (Figure 3C).

### Effects of Low Salinity on Apoptosis-Related Genes Expression

**Figure 4:**
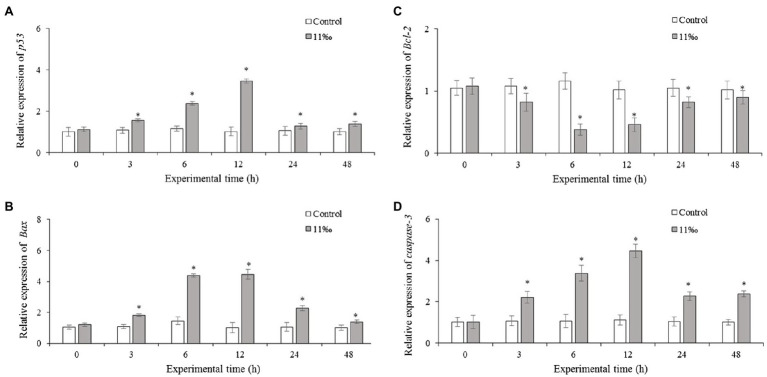
Relative mRNA expression levels of *Ptp53* **(A)**, *Bcl-2* **(B)**, *Bax* **(C)**, and *caspase-3* **(D)** in the gills of *P. trituberculatus* under low salinity stress (*n*=6). Results that are significantly different from the control are indicated using asterisks (*p*<0.05). Bcl-2, BCL2 apoptosis regulator; Bax, BCL2-associated X apoptosis regulator.

To investigate the cell apoptosis effect of p53 on *P. trituberculatus* under salinity stress, the mRNA expression of p53 regulated genes was examined. Results showed that *Ptp53* mRNA expression in the low salinity group increased significantly from 3 to 24h compared with that in the control group (Figure 4A). Similarly, *Bax* mRNA expression increased significantly from 3 to 48h of low salinity exposure compared with that in the control group (Figure 4B), and *Bcl-2* mRNA expression showed roughly opposite trend to that of *Bax*; *Bcl-2* mRNA expression in the low salinity group was reduced compared with that in the control group (Figure 4C). *Caspase-3* mRNA expression increased significantly from 3 to 24h of low salinity stress compared with that in the control group (Figure 4D).

### Western Blotting

**Figure 5:**
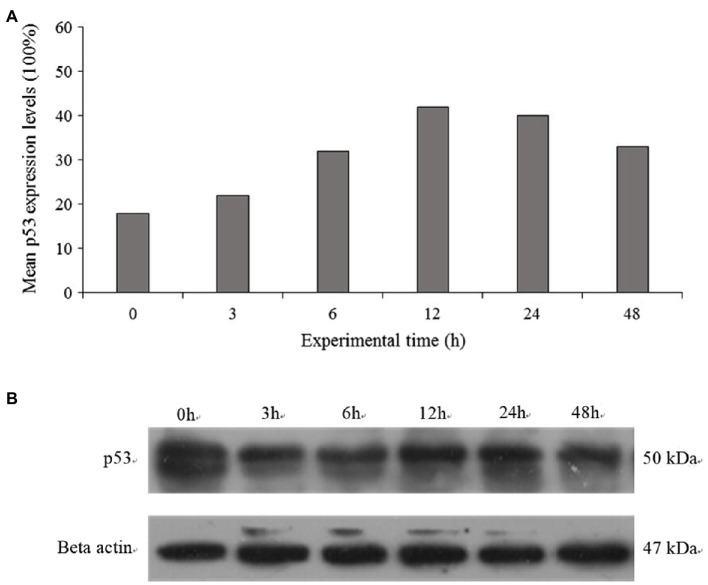
The protein expression of p53 was detected by Western blotting at 0h, 3h, 6h, 12h, 24h, and 48h **(A)**. Quantitative analysis results of p53 was presented as the ratio of band density to that of β-actin **(B)**.

Western blot was used to investigate the Ptp53 protein levels in the gills of crab. As shown in Figure 5A, Western blot analysis revealed a p53 protein band with an apparent molecular weights of approximately 53kDa. Salinity treatment induced the expression of Ptp53 in the gills, with expression increasing with salinity (Figure 5B). Ptp53 expression was significantly higher in the 11ppt treatment group than in the control group (*p*<0.05).

### Effects of Ptp53-Interfered on the Mortality of *P. trituberculatus* After Low Salinity

**Figure 6:**
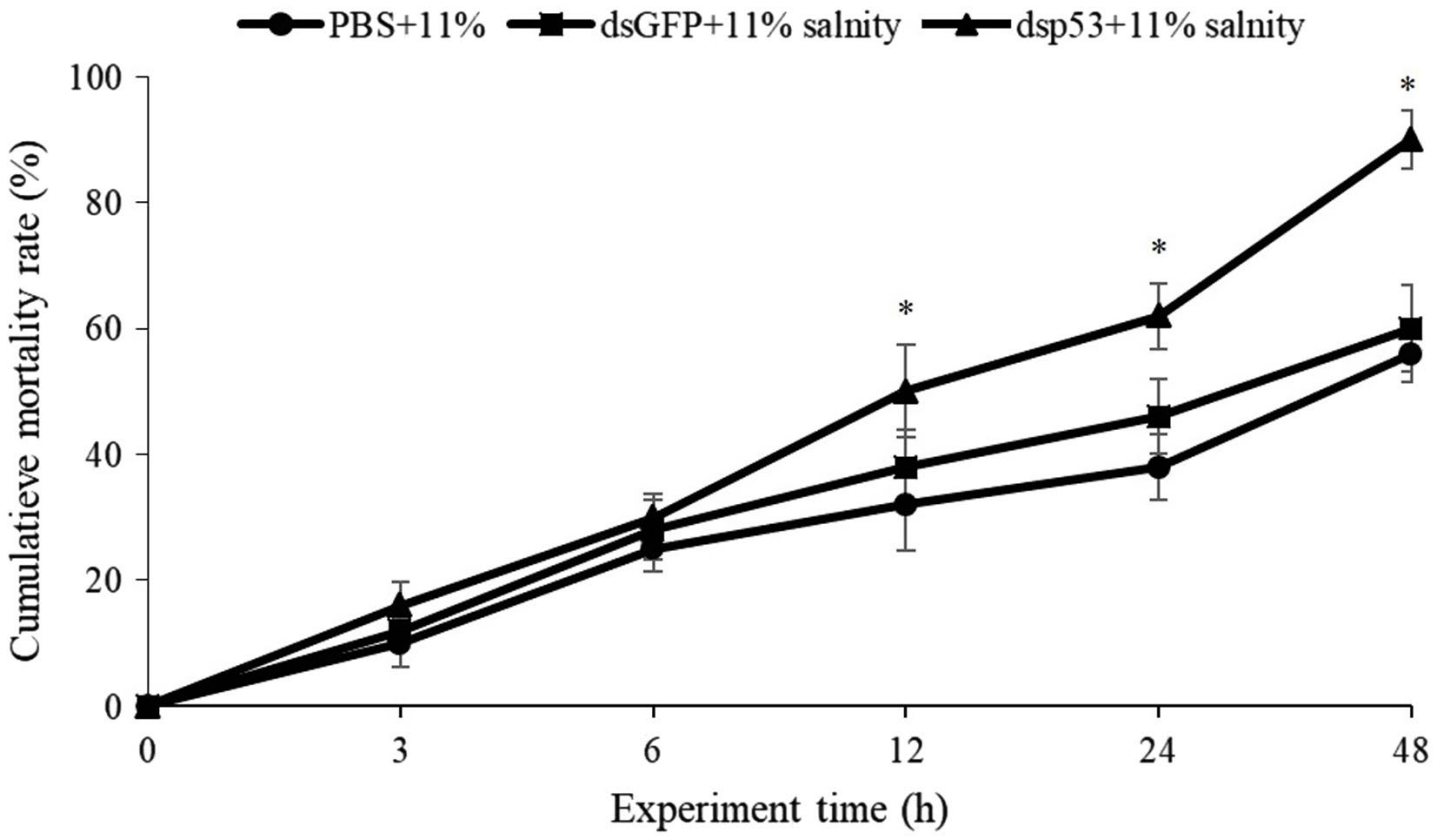
Cumulative mortality of *P. trituberculatus* in the PBS, dsGFP, and dsp53 groups (dsGFP and PBS groups were the controls) under low salinity stress. Significant differences between the dsp53 and dsGFP groups at the same time point are indicated using asterisks. PBS, phosphate-buffered saline; GFP, green fluorescent protein.

Figure 6 shows the cumulative mortality of *P. trituberculatus* in the dsPtp53, dsGFP, and PBS groups under low salinity conditions. Among the crabs in the dsPtp53 group, mortality increased markedly from 6 to 48h, and their cumulative mortality at 24h and 48h was significantly higher than those in the dsGFP and PBS groups. The mortality rates at 48h were 56% in the PBS group, 60% in the dsGFP group, and 92% in the dsPtp53 group.

## Discussion

The biochemical and physiological mechanisms of *P. trituberculatus* salinity tolerance have been studied widely (Pan et al., 2016; Wang et al., 2018). Previously, we demonstrated that antioxidant defense in *P. trituberculatus* is enhanced under conditions of salinity stress (Wang et al., 2018). However, the gene expression and signaling changes in response to salinity stress in *P. trituberculatus* are unknown. P53 is a major regulatory factor of cell metabolism (Liang et al., 2013). Various stress responses, such as DNA damage and hypoxia, are believed to involve in p53 signaling pathway (Krieg et al., 2006; Ching et al., 2013).

In the present study, we cloned a *p53* gene from *P. trituberculatus*. The putative Ptp53 protein had characteristic features of p53 proteins, such as a DNA-binding site and a zinc finger motif. BLAST analysis showed that the protein encoded by *Ptp53* of has high homology with other invertebrate p53 proteins (50–56%). The high similarities of the Ptp53 protein with those of *P. vannamei* and *Eriocheir sinensis* suggest similar regulatory roles of p53 in these species. The conserved amino acid residues involved in DNA and zinc binding among multiple species suggest the essential functions of these residues in *p53* (Das et al., 2019). The phylogenetic tree analysis indicated that the structure of p53 is highly conserved and that p53 is highly conserved among crustaceans (Ching et al., 2013). qRT-PCR detected ubiquitous expression of *Ptp53* in all tested tissues, with the gills showing the highest expression level. These findings implied that Ptp53 has important functions under low salinity stress in *P. trituberculatus*.

In the cellular defense against xenobiotic stress, antioxidant enzymes (GPX, CAT, and SOD) have vital functions (Sevcikova et al., 2011). SOD catalyzes superoxide anion radicals and converts oxygen-free radicals to hydrogen peroxide, thus balancing free radical metabolism and protecting cells from damage (Filho, 2007; Cao et al., 2010). CAT and GPX have the ability to eliminate and transform H_2_O_2_ into H_2_O and O_2_, thereby reducing tissue injury (Yonar et al., 2012). These enzymes represent an organism’s first line of defense against stress caused by toxin exposure, and their activities are required to prevent cell damage or death (Pandey et al., 2008). Herein, we observed that low salinity conditions increased the mRNA expression and activities of GPX, CAT, and SOD. This response is likely to represent a defense mechanism to resist increased ROS levels. SOD increases or stimulation enhances the H_2_O_2_ concentration, which in turn is eliminated by CAT. An excess of salinity could ultimately damage antioxidant enzyme functions, resulting in reduced SOD activity.

Here, for the first time, we report that the *p53* mRNA expression and p53 protein levels in the gills of *P. trituberculatus* are increased in response to low salinity conditions. Similarly, a significant increase in p53 mRNA and protein levels was observed in *Anabas testudineus* and *Trachemys scripta elegans* under low salinity stress (Ching et al., 2013; Li et al., 2019), suggesting that apoptotic signals appear early during the progressive acclimatization to low salinity conditions. The cellular apoptosis level will increase after external stimuli. To assess p53 transcriptional activation under low salinity, specific downstream regulatory targets: Bax, Bcl-2, and caspase-3 were assessed (Zeng et al., 2014). The pro-apoptotic Bcl-2 family member Bax is located in the mitochondrial outer membrane, and its transcription is activated directly by p53 (Jin et al., 2011). Bax induces cytochrome c release into the cytosol, while the anti-apoptotic factor Bcl-2 inhibits cytochrome c release from mitochondria. Thus, the release of cytochrome c is affected by the intracellular Bcl-2: Bax ratio (Zhang et al., 2020). Caspase-3 activation results in the cleavage of a series of proteins, ultimately leading to apoptosis (Mardones and Escarate, 2014). Herein, low salinity resulted in significantly increased Bax levels and decreased Bcl-2 levels in the gills, which suggested that salinity stress might induce apoptosis *via* the cytochrome c pathway in a time and tissue-dependent manner. These results further suggested that the p53-Bax-Bcl-2-caspase axis is involved in the apoptosis of *P. trituberculatus* induced by salinity. Similar results were found in the white shrimp *Litopenaeus vannamei*, in which silencing of *p53* decreased the expression of caspase-3 after 48h of hypoxia; however, it increased caspase activity under normoxic conditions (Nuñez-Hernandez et al., 2018).

The membrane expression of death receptors is regulated non-transcriptionally and transcriptionally by p53, and p53-mediated apoptosis proceeds *via* effector caspase activation or death receptor signaling (the extrinsic pathway; Schuler and Green, 2001). Earlier research suggested that low salinity activated p53, which induced apoptosis (Pourmozaffar et al., 2020). Knockdown of *Ptp53* increased the mortality of *P. trituberculatus* significantly under low salinity stress. This suggested that knockdown of *Ptp53* inhibited antioxidant defense and the capacity to repair DNA under low salinity conditions. Collectively, our findings implied an important role of Ptp53 in *P. trituberculatus* under low salinity stress.

## Conclusion

The findings of our study revealed the function of Ptp53 in response to low salinity stress. Ptp53 showed the highest expression in the gills. Under low salinity stress, gill mRNA and protein levels of Ptp53 increase in *P. trituberculatus*. Low salinity stress produced oxidative stress, leading to apoptosis. In addition, *Ptp53* knockdown increased crab mortality significantly under low salinity conditions. Further research is needed to explain the mechanism of p53 functions in response to environmental stress and to clarify the role of the apoptotic pathway in *P. trituberculatus*.

## Data Availability Statement

The original contributions presented in the study are included in the article/Supplementary Material, and further inquiries can be directed to the corresponding author.

## Author Contributions

XR: investigation, writing – original draft, and funding acquisition. LW: formal analysis and funding acquisition. YX: methodology and funding acquisition. QW: visualization. JLv: writing – review and editing. PL and JLi: conceptualization, methodology, supervision, and funding acquisition. All authors contributed to the article and approved the submitted version.

## Funding

This work was supported by the National Natural Science Foundation of China (grant numbers 41876186 and 41776160), the China Agriculture Research System (grant number CARS-48), and the Basic Scientific Research Business Expenses of Chinese Academy of Fishery Sciences of “Innovation team project of ecological aquaculture in seawater pond” (grant number 2020td46).

## Conflict of Interest

The authors declare that the research was conducted in the absence of any commercial or financial relationships that could be construed as a potential conflict of interest.

## Publisher’s Note

All claims expressed in this article are solely those of the authors and do not necessarily represent those of their affiliated organizations, or those of the publisher, the editors and the reviewers. Any product that may be evaluated in this article, or claim that may be made by its manufacturer, is not guaranteed or endorsed by the publisher.

## Footnotes

^1^ https://blast.ncbi.nlm.nih.gov/Blast.cgi

^2^http://smart.embl-heidelberg.de/.

^3^https://www.ncbi.nlm.nih.gov/orffinder/.

^4^ https://web.expasy.org/compute_pi/

^5^http://tcoffee.crg.cat/apps/ampa/do.

^6^ http://www.cbs.dtu.dk/services/SignalP/

## Supplementary Material

The Supplementary Material for this article can be found at: https://www.frontiersin.org/articles/10.3389/fphys.2021.724693/full#supplementary-material
