# Supplementary material for: Characterization of p53 From the Marine Crab Portunus trituberculatus and Its Functions Under Low Salinity Conditions
Source: Front Physiol. 2021 Oct 21;12:724693. doi: 10.3389/fphys.2021.724693 (PMC8568311; doi:10.3389/fphys.2021.724693)
Supplement: Supplementary file 1 [file Data_Sheet_1.DOCX]

**Figure S1**


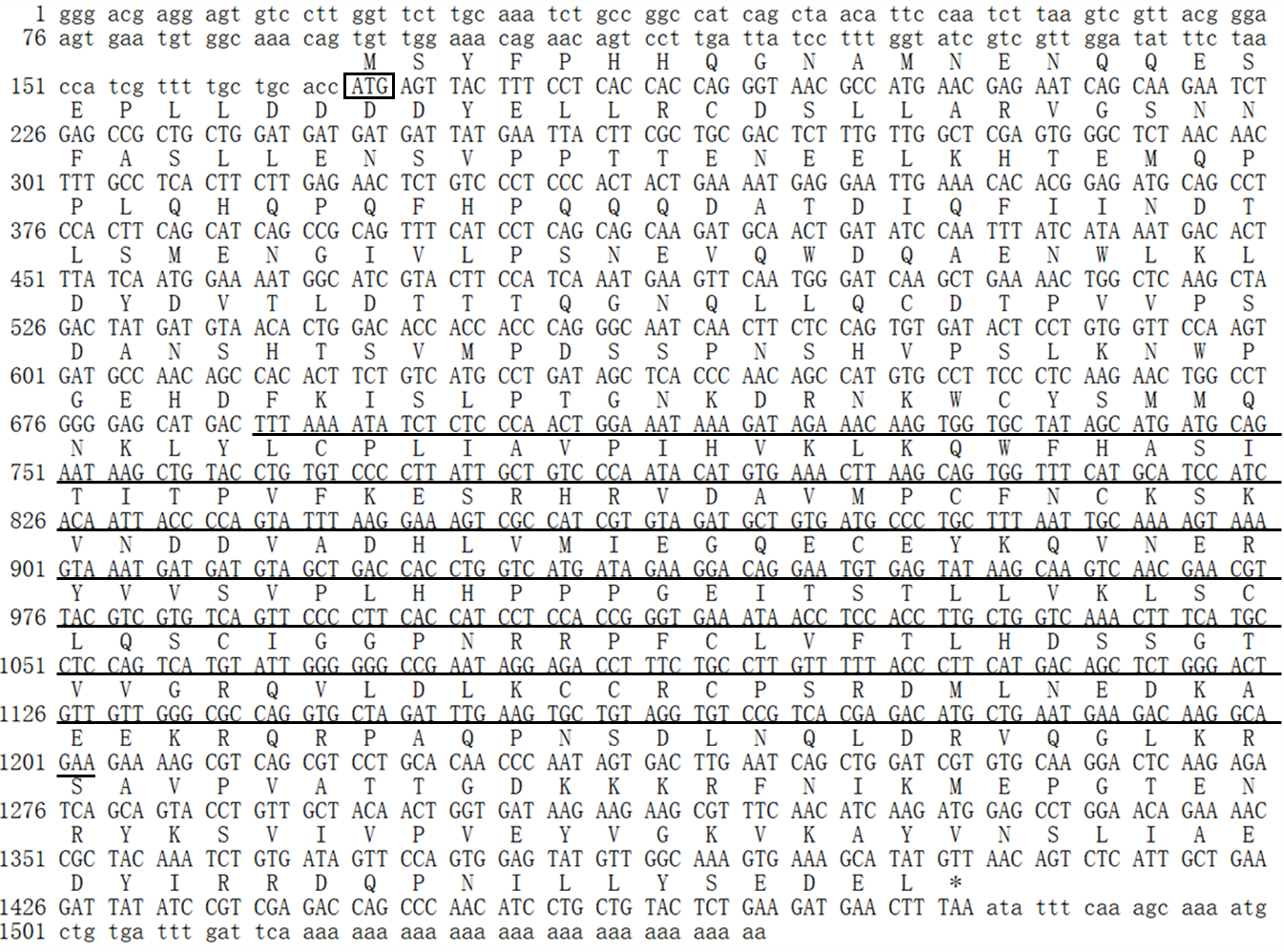


**Figure S2**


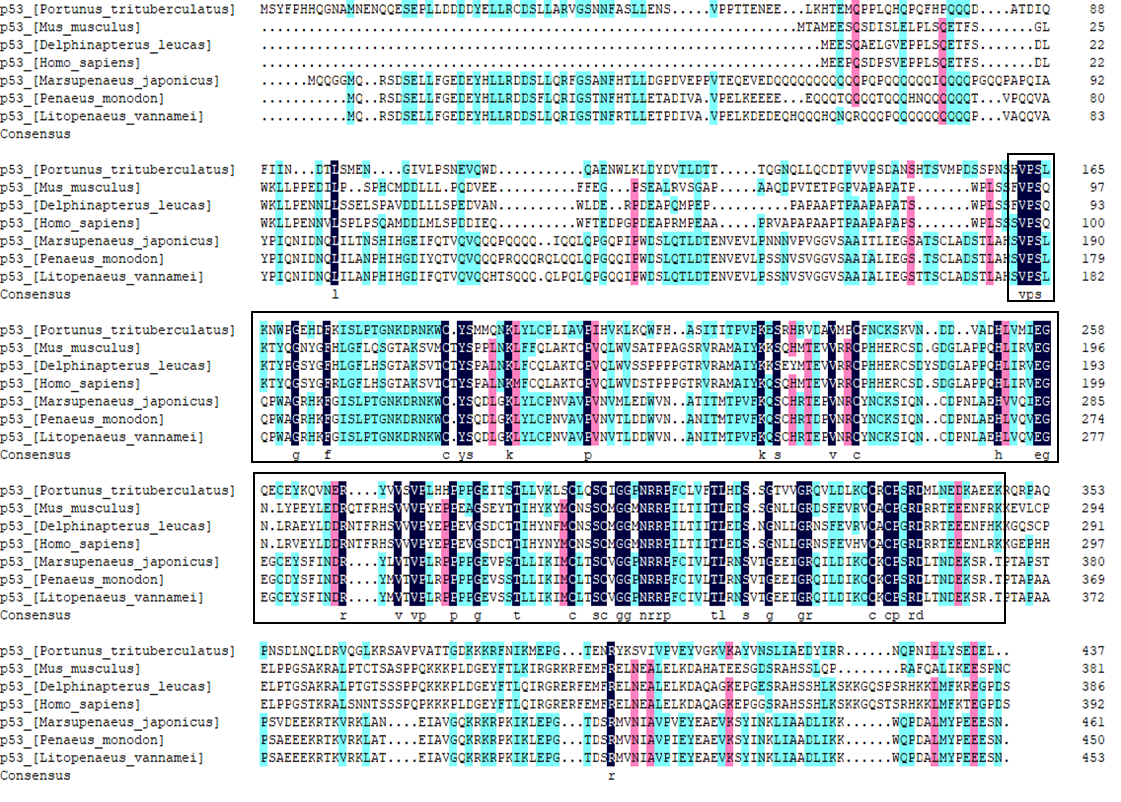


**Figure S3**


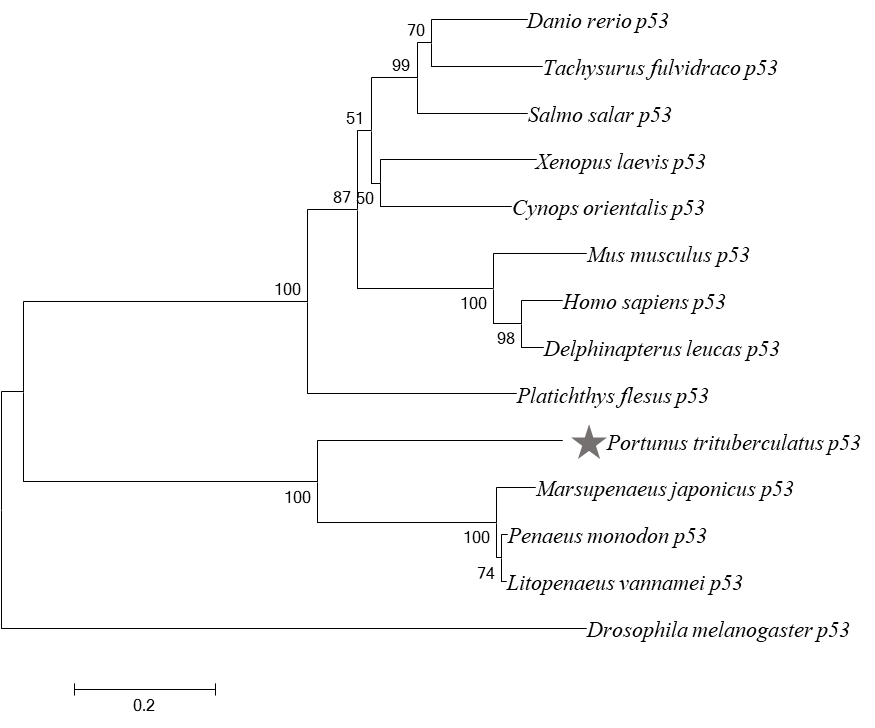


**Figure S4**


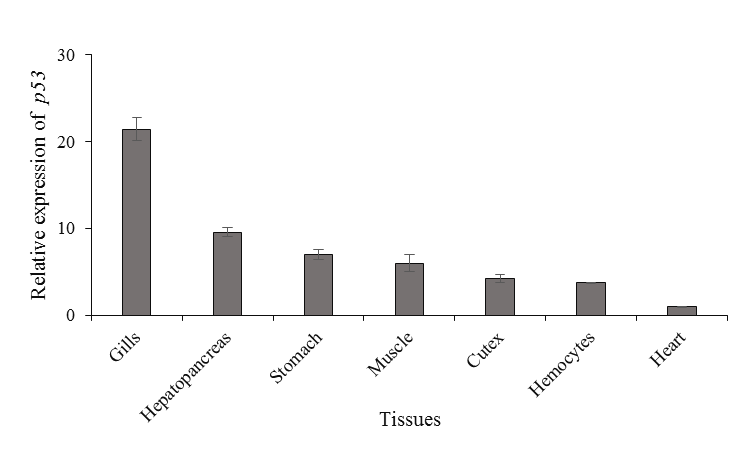


**Figure Captions**

**Fig.** **S1** The nucleotide sequence and deduced amino acid sequence of *p53* from *P. trituberculatus*.

The initiation codon (ATG) is marked with a black box. The asterisk indicates the termination codon (TGA). The conserved domain of p53 is underlined.

**Fig. S2** Multiple alignment of the amino acid sequences of p53 from *P. trituberculatus* and other species.

**Fig. S3** Phylogenetic tree constructed using the neighbor-joining method for p53 from *P. trituberculatus* and other species.

**Fig. S4** Distribution of *p53* expression in different tissues of *P. trituberculatus.* The data are the mean ± SD values of six separate individuals.
